# Tricuspid Valve Regurgitation after Orthotopic Heart Transplantation: Prevalence and Etiology

**DOI:** 10.1155/2012/120702

**Published:** 2012-10-14

**Authors:** Yaniv Berger, Yedael Har Zahav, Yigal Kassif, Alexander Kogan, Rafael Kuperstein, Dov Freimark, Jacob Lavee

**Affiliations:** ^1^Heart Transplantation Unit, Department of Cardiac Surgery, Leviev Heart Center, Sheba Medical Center, and the Sackler Faculty of Medicine, Tel Aviv University, 52621 Ramat Gan, Israel; ^2^Heart Institute, Leviev Heart Center, Sheba Medical Center, and the Sackler Faculty of Medicine, Tel Aviv University, 52621 Ramat Gan, Israel

## Abstract

*Background*. Tricuspid valve regurgitation (TR) after orthotopic heart transplantation (OHT) is common. The aims of this study were to determine the prevalence of TR after OHT, to examine the correlation between its development and various variables, and to determine its outcomes. *Methods*. All 163 OHT patients who were followed up between 1988 and 2009 for a minimal period of 12 months were divided into those with no TR/mild TR and those with at least mild-moderate TR, as assessed by doppler echocardiography. These groups were compared regarding preoperative hemodynamic variables, surgical technique employed, number of endomyocardial biopsies, number of acute cellular rejections, incidence of graft vasculopathy, and clinical outcomes. *Results*. At the end of the followup (average 8.2 years) significant TR was evident in 14.1% of the patients. The development of late TR was found by univariate, but not multivariate, analysis to be significantly correlated with the biatrial surgical technique (*P* < 0.01) and the presence of graft vasculopathy (*P* < 0.001). TR development was found to be correlated with the need for tricuspid valve surgery but not with an increased mortality. *Conclusions*. The development of TR after OHT may be related to the biatrial anastomosis technique and to graft vasculopathy.

## 1. Introduction

Tricuspid regurgitation (TR) after orthotopic heart transplantation (OHT) is common, with reported prevalence that varies from 19% to 84% [1]. The prevalence and severity of TR increase with the length of followup. In most cases TR is mild and asymptomatic, but some cases of moderate or severe TR are related to morbidity and mortality [1–5]. Doppler echocardiography is the most common technique used for the detection and evaluation of severity of TR [6, 7]. The treatment of severe symptomatic TR is mainly conservative with diuretics. In refractory cases there is an indication for tricuspid valve repair or replacement surgery. The etiology of TR after OHT is unclear, and several variables have been reported to be related, including the surgical anastomosis technique employed (biatrial versus bicaval) [8–13], iatrogenic damage due to endomyocardial biopsies (EMBs) [8, 14–18], number of acute cellular rejection episodes (ACRs) [8, 14], pretransplant pulmonary hypertension [8, 19, 20], discordance between the size of the donor's heart and the recipient's pericardial cavity [21], and cardiac allograft vasculopathy (CAV) [14]. Preventive measures mentioned in the literature include prophylactic tricuspid annuloplasty during OHT [22–24], the use of a long bioptome sheath during EMB [18], and the use of noninvasive methods to monitor for graft rejections [25].

The aims of this study were to determine the short and long term prevalence of TR after OHT, to examine the correlation between its development and the above-mentioned variables, and to determine its clinical outcomes.

## 2. Material and Methods

The study is a retrospective cohort study of all 163 patients who underwent OHT between 1988 and 2009 and were followedup at the heart transplant clinic at the Sheba Medical Center for a minimal period of 12 months. The data of 10 aditional patients who underwent heart transplantations in China between 2005 and 2007 was excluded in accordance with current ethical guidelines of transplantation societies and journals. Patients were divided into two groups based on the severity of their postoperative TR at the end of the follow-up period at an average of 8.2 years: 140 patients with no TR or mild TR and 23 patients with significant (at least mild to moderate) TR. The two groups were compared regarding preoperative hemodynamic variables, surgical technique employed (biatrial versus bicaval anastomosis), number of EMBs taken, number of ACRs, incidence of CAV, right heart failure, and clinical outcome. 

### 2.1. Evaluation of TR

Patients underwent routine echocardiographic follow-up examinations immediately after transplantation, at 3 months, 12 months, and annually thereafter. Severity of TR was assessed by comparing the ratio of TR jet area to the right atrial area on color Doppler and was scored from 0 to 3 : 0—no TR, 1—mild TR, 2—moderate TR, and 3—severe TR. Mild to moderate TR was scored 1.5 and moderate to severe TR was scored 2.5. Additional echocardiographic parameters reviewed included left ventricular ejection fraction, right ventricular size and function, and estimated pulmonary arterial pressure.

### 2.2. Invasive Evaluation

Every patient underwent routine right heart catheterization before transplantation, one week after transplantation and annually thereafter in which mean pulmonary arterial pressure, pulmonary vascular resistance, and right atrial pressure were recorded. The incidence of CAV was evaluated by annual coronary angiography.

EMBs were performed weekly during the first month after transplantation, biweekly during the second and third months, at the fourth, fifth, sixth, ninth, and twelfth months after transplantation, and annually following the first year. Further biopsies were performed when clinical suspicion of graft rejection was raised. Biopsies were evaluated for rejection using the revised ISHLT (International Society of Heart and Lung Transplantation) criteria [26].

### 2.3. Statistical Analysis

Patient data were analyzed with the Statistical Program of Social Sciences (SPSS version 19). Categorical data are expressed as percentages and continuous data are expressed as mean ± standard deviation. The prevalence of late irreversible TR and the survival difference between the two study groups were analysed according to the Kaplan-Meier method. Univariate analyses were used to compare differences between variables obtained in the two groups of patients (patients with no/mild TR versus patients with at least mild-moderate TR). We have conducted a nonparametric Kolmogorov-Smirnov test for normal distribution; based on the results, we used independent *t*-test or Mann-Whitney test to compare continuous data (number of EMBs, number of ACRs, and preoperative hemodynamic variables) between the two groups; we presented median (and range) for variables which depart from normal distribution. Categorical data (CAV, surgical anastomosis technique and mortality rates) were compared by *χ* ^2^ test. A multivariate analysis was performed by using the Cox regression model to identify risk factors for TR. A *P* value of less than 0.05 was defined as significant. 

## 3. Results

### 3.1. Patients Demographics and Perioperative Data

There were no statistically significant differences between the two groups in the following preoperative demographic or clinical characteristics: gender, age, etiology of heart failure, or incidence of previous heart surgery (Table 1). Of the total 163 patients, 55.8% underwent the transplant at the Sheba Medical Center in Israel and the rest in other countries, with a statistically significant difference between the two groups (Table 1). The average total heart ischemic time during transplantation, as well as the average cardiopulmonary bypass time, was similar between the two groups (Table 1). The average follow-up period was significantly longer in the group of patients with significant TR compared to the group of patients with insignificant TR (Table 1).

### 3.2. TR Prevalence

**Figure 1:**
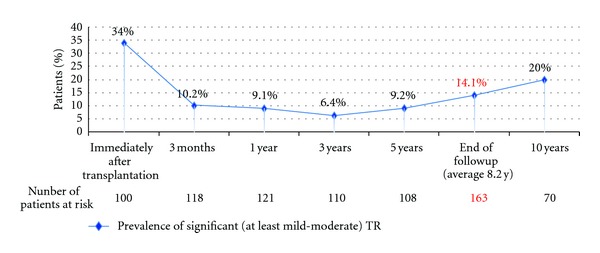
Prevalence of significant (at least mild-moderate) tricuspid regurgitation (TR) at different time points.

Significant TR prevalence peaked at 34% immediately after transplantation (average 14.0 ± 11.8 days), decreased to a nadir of 6.4% after 3 years, and then increased gradually (Figure 1). At the end of the followup, 23 patients (14.1% of the study population) developed significant TR. The average length of time between transplantation and the development of significant irreversible TR was 6.8 ± 4.8 years. 

**Figure 2:**
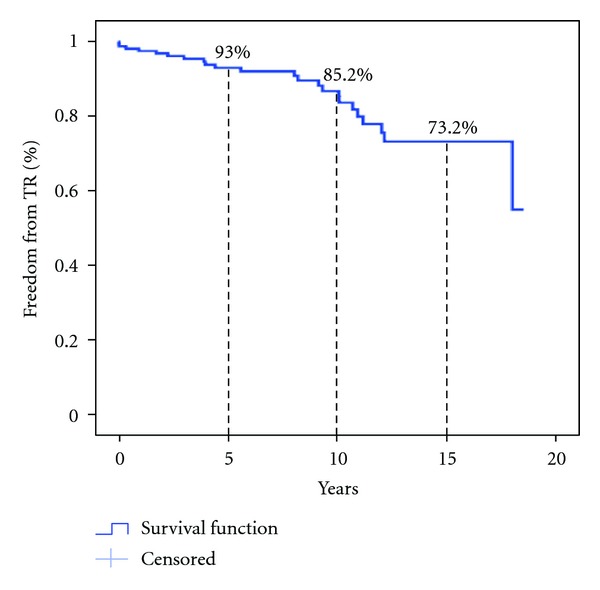
Kaplan-Meier analysis of freedom from late significant (at least mild-moderate) tricuspid regurgitation (TR).

A Kaplan-Meier analysis of freedom from late significant TR revealed that 10 years after transplant 85.2% were permanently free of significant TR (Figure 2). 

### 3.3. Risk Factors for TR

Univariate analysis of risk factors for early (average 14.0 ± 11.8 days) development of significant TR (Table 2) yielded only higher values of pretransplant mean pulmonary arterial pressure. The relationship between early TR and pretransplant pulmonary vascular resistance was of borderline significance. The surgical technique—biatrial or bicaval anastomosis—did not have an impact on the development of early TR. 

Univariate analysis of risk factors for the late development of significant TR at the end of the followup is depicted in Table 3. The average total number of EMBs taken during the follow-up period was significantly higher in the group that developed significant TR at the end of the follow-up period; however the average number of EMBs taken prior to the development of significant TR was similar in both groups. 

The median of ACR episodes with any grade equal or higher than ISHLT grade 1R was similar in both groups, as well as the median of ACR episodes with ISHLT grade 2R or higher. 

Hemodynamically, the significant TR group displayed higher pretransplant values of mean pulmonary arterial pressure and pulmonary vascular resistance compared with the no/mild TR group, and these differences were of borderline significance (*P* = 0.064 and *P* = 0.070, resp.).

The bicaval surgical anastomosis technique was employed in 55.8% of the patients and the biatrial technique was employed in 35%; in 9.2% of the patients (all of them underwent OHT in foreign countries) the surgical technique employed could not be determined due to missing surgery reports. The proportion of patients who underwent OHT by using the biatrial surgical technique was significantly higher in the significant TR group (65% compared with 34.4% in the no/mild TR group).

During the follow-up period 60 patients of the study population (39.7%) developed CAV of any kind (including insignificant disease of one or more coronary arteries). Significant CAV (defined as significant stenosis of at least one coronary vessel) was found in 48 patients (31.8%) and two or three vessels CAV was diagnosed in 36 patients (23.8%). The prevalence of CAV in the significant TR group was higher compared with the no/mild TR group (Table 3).

In multivariate analysis none of the explored variables were found to be an independent risk factor for the development of late significant TR. The explored variables included total number of EMBs taken, total number of ACRs with at least ISHLT grade 1R, the surgical technique employed, and incidence of CAV.

### 3.4. Clinical Outcomes of TR

**Figure 3:**
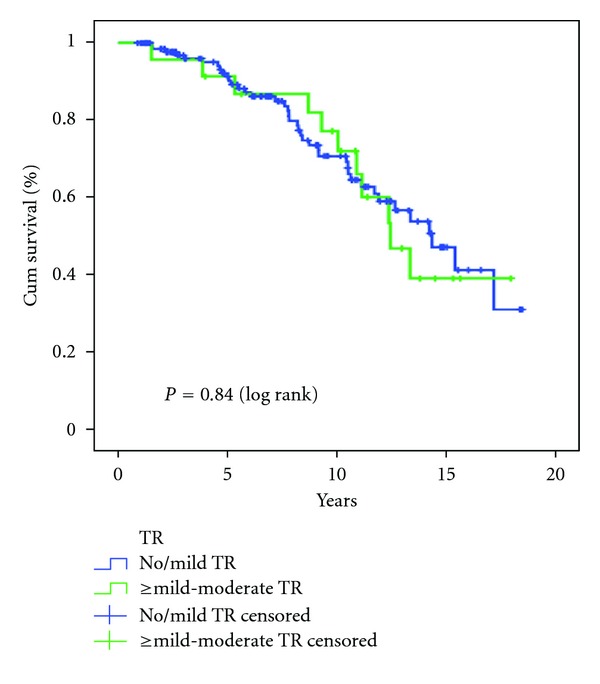
Kaplan-Meier survival analysis of both study groups. TR, tricuspid regurgitation.

The relationship between significant TR at the end of the follow-up period and clinical outcomes is depicted in Table 4. The total mortality rate over the follow-up period (average 8.2 years) was 31.3%. In univariate analysis, the mortality rate during the follow up period was higher in the significant TR group (47.8%) compared with the no/mild TR group (28.6%), and this difference was of borderline significance (*P* = 0.065). A Kaplan-Meier survival analysis did not demonstrate a significant difference between groups (Figure 3). 

No significant differences were observed between the two groups in the median serum creatinine level at the end of the followup (Table 4). 

During the follow-up period 8 patients underwent repeat heart surgery: 6 patients in the significant TR group and two patients in the no/mild TR group. Of the six patients in the significant TR group, one patient underwent tricuspid annuloplasty (8.7 months after OHT), two patients underwent tricuspid valve replacement (1.0 and 13.4 years after OHT), two patients underwent combined replacement of the tricuspid and mitral valves (5.3 and 8.8 years after OHT), and one patient underwent superior vena cava thrombectomy. In the no/mild TR group one patient underwent implantation of biventricular assist device as bridge to retransplantation and one patient underwent coronary artery bypass grafting. 

**Figure 4:**
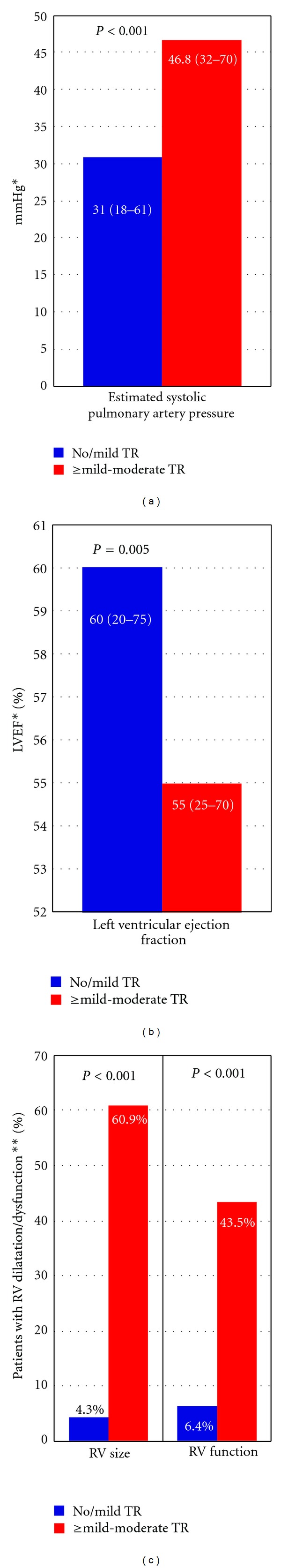
Relationship between late TR severity and echocardiographic parameters TR, tricuspid regurgitation; LVEF, left ventricular ejection fraction; RV, right ventricle.*Data are presented as median and range (min–max). **% Patients with right ventricular dilatation/dysfunction ≥mild.

The relationship between the development of significant TR at the end of the follow-up period and echocardiographic parameters (as measured in the last echocardiographic exam) is depicted in Figure 4. Patients in the significant TR group showed significantly higher values of estimated systolic pulmonary artery pressure, lower left ventricular ejection fraction, increased right ventricular dilatation, and worse levels of right ventricular dysfunction compared to the no/mild TR group. 

## 4. Discussion

TR after OHT is a common problem, varying in prevalence between 5.5% and 54% (Table 5). Although comparing different series may be problematic because studies vary in the length of followup, in the definition of significant TR and in the technique used for TR diagnosis, all reported series except one [25] demonstrated a higher prevalence of TR at the end of the followup in comparison to our study (14.1%). Marelli et al. [4] in a cohort of 670 patients found freedom from significant TR of 78% at 9 years. Our analysis demonstrated a slightly higher rate of 85.2% at 10 years (Figure 2), similar to the 85.8% at 10 years rate demonstrated by Chan et al. [3].

Regardless of the incidence of its occurrence, all reported series [2, 4, 5, 14] have demonstrated an increased mortality among patients who have developed postoperative significant TR, ranging from 15% to 62.5%. Similarly, while the overall mortality rate during the follow-up period (average 8.2 ± 4.6 years) in our study was 31.3%, similar to the reported ISHLT registry data [29], the mortality rate in the significant TR group was higher compared with the no/mild TR group (47.8% versus 28.6%), and this difference was of borderline significance (*P* = 0.065); the Kaplan-Meier survival analysis did not demonstrate a significant difference between the two groups in our study. Moreover, the development of significant TR is associated with the significant morbidity of the right heart failure and with the increased necessity to undergo repeat operations to repair or replace the regurgitant tricuspid valve, as evident in our series. Thus it is of paramount importance to identify the risk factors associated with this potentially lethal complication in order to try and avoid them. 

The etiology of postoperative TR after OHT is no doubt multifactorial and several preoperative, intraoperative, and postoperative features have been implicated as potential causative factors. Several studies have reported a correlation between high pretransplant pulmonary vascular resistance or pulmonary arterial pressure and development of TR [8, 19, 20]. The suggested mechanism is the postsurgical right ventricular dysfunction and dilatation due to increased afterload. Our study has demonstrated a borderline significant correlation between high pretransplant values of pulmonary arterial pressure and pulmonary vascular resistance and the development of late TR (Table 3); we have also found a statistically significant correlation between high pretransplant values of pulmonary arterial pressure and early TR (Table 2), implying that elevated pulmonary arterial pressure and resistance may be risk factors for developing early and late TR after OHT. 

Many studies have found a correlation between the biatrial surgical technique and the development of TR after OHT [8, 10–13]. The suggested mechanism in this case is geometric distortion of the atrioventricular annular ring and malcoaptation of the valve leaflets. Our study has also demonstrated this correlation with patients who underwent the biatrial anastomosis technique showing significantly higher incidence of late significant TR compared to patients who underwent the bicaval anastomosis (Table 3). The lack of significant difference in our series in the incidence of significant TR between the two anastomosis techniques early after the operation could be attributed to the residual high pulmonary artery pressures and resistance in the immediate postoperative period which resolves over time. 

Many studies point to a possible relationship between EMBs and the development of TR. Nguyen et al. [16] reported a TR prevalence of 60% at the end of the followup among patients who underwent more than 31 EMBs compared to 0% among patients who underwent less than 18 EMBs and in a multivariate analysis found that only the total number of EMBs was an independent risk factor for TR severity. Lo et al. [15] have demonstrated correlation between EMBs number greater than 10 and iatrogenic damage to the tricuspid valve. Our study has demonstrated no correlation between the average number of EMBs taken prior to the development of significant TR, and in fact this number was actually higher in the no/mild TR group (Table 2). The increased total number of EMBs found in the significant TR group in our study simply reflects the longer follow-up period of the significant TR group. Among our total study population, only two patients were found by echocardiography to have developed iatrogenic damage to the tricuspid valve during the follow-up period, as evident by flail tricuspid leaflet in both cases and only one of these patients developed significant TR. 

A few studies have found a correlation between the number of ACRs and TR. Aziz et al. [8] have shown a statistically significant relationship between ACRs number greater than or equal to ISHLT grade 2 and early and late TR. Hausen et al. [14] have demonstrated a correlation between TR and the number of ACRs during the first postoperative year. In our study late TR was not found to be correlated with the number of ACRs equal or greater than ISHLT grade 1R, and there was also no difference in the number of ACRs with grade 2R or higher among the two groups (Table 3). 

The relationship between CAV and the development of TR has not been reported in previous studies and only one study [14] has found such a relationship. Our study has demonstrated a strong link in univariate analysis between CAV and late TR (Table 3) but no such link in multivariate analysis. As CAV is a well-known complication of the long-term course after transplantation, it is unclear whether this link with late TR is causative or purely incidental and time dependent. At the end of the followup the measured left ventricular ejection fraction in our series was lower in the significant TR group compared to the group with no TR. This unexpected finding, together with the increased levels of right ventricular dilatation and dysfunction (Figure 4), might hint to a common etiology of graft vasculopathy.

Our study has several limitations. The study is retrospective, and therefore some data regarding early postoperative parameters were missing, particularly for patients transplanted in foreign countries. The study population is heterogenous due to this retrospective design—some patients underwent OHT 20 years before other patients, so different patients were exposed to different treatment protocols. The grading of TR was assessed by measuring the size of regurgitant jet area on color Doppler—this technique, although used in other similar studies, is not quantitative. The average follow-up period of the significant TR group was significantly longer compared with the insignificant TR group, and as TR prevalence increases with time, this difference between groups can act as a confounder; this difference can also explain the similar survival curves of both groups (Figure 3) despite the higher mortality rate among patients with significant TR. 

In conclusion, the results of our series have demonstrated that the development of TR after OHT is probably related to pretransplantation increased pulmonary artery pressure and pulmonary vascular resistance, biatrial anastomosis technique, and maybe to the development of graft vasculopathy. It is probably not related to the total number of EMBs and the number of ACRs. The development of TR is probably associated with increased mortality but definitely with the need for a repeat tricuspid surgery.

## Figures and Tables

**Table 1:** Patients demographics and characteristics.

		No TR/mild TR *n* = 140	≥mild-moderate TR *n* = 23	*P* value
Gender (% males)		82.8%	91.3%	NS
Age		50.0 ± 13.3	50.0 ± 12.6	NS

	ICM	52.9%	56.5%	
Etiology	DCM	18.6%	21.7%	NS
	Other	28.6%	21.7%	

Place of transplantation	Israel	59.3%	34.8%	0.028

Previous heart surgery		41.2%	35.3%	NS
Total ischemic time (minutes)		160.5 ± 52.1	170.8 ± 52.2	NS
Bypass time (minutes)		148.8 ± 34.2	150.0 ± 49.9	NS
Followup (years)		7.8 ± 4.6	10.5 ± 4.1	0.009

TR: tricuspid regurgitation; ICM: ischemic cardiomyopathy; DCM: dilated cardiomyopathy; NS: nonsignificant.

**Table 2:** A univariate analysis of risk factors for early significant (at least mild-moderate) TR.

Risk factor		No TR/mild TR (*n*)	At least mild-moderate TR (*n*)	*P* value
	Average pretransplant MPAP (mmHg)	34.0 ± 15.4 (*n* = 37*)	43.0 ± 10.8 (*n* = 24*)	0.009
Pre-transplant hemodynamic parameters	Average pre-transplant PVR (Wood units)	2.9 ± 2.2 (*n* = 37*)	4.1 ± 2.3 (*n* = 23*)	0.066
	Average pre-transplant right atrial pressure (mmHg)	10.5 ± 6.3 (*n* = 31*)	13.2 ± 6.1 (*n* = 18*)	0.141

Surgical technique employed	Biatrial %	10.2% (*n* = 6/59*)	8.8% (*n* = 3/34*)	0.833

TR: tricuspid regurgitation; MPAP: mean pulmonary arterial pressure; PVR: pulmonary vascular resistance.

*Relatively small numbers of patients due to missing early postoperative data, particularly for patients transplanted in foreign countries.

**Table 3:** A univariate analysis of risk factors for late significant (at least mild-moderate) TR.

Risk factor		No TR/mild TR *n* = 140	At least mild-moderate TR n = 23	P value
Number of EMBs	Average total number of EMBs taken	17.5 ± 5.9	20.7 ± 6.5	0.045
	Average number of EMBs taken before TR development	17.5 ± 5.9	15.7 ± 9.1	0.431

Number of ACRs*	Median number of ACRs ≥ grade 1R**	6 (0–24)	7 (2–23)	0.087
	Median number of ACRs ≥ grade 2R**	0 (0–7)	1 (0–8)	0.712

	Average MPAP (mmHg)	36.8 ± 13.7	47.0 ± 12.9	0.064
Pre-transplant hemodynamic parameters	Average PVR (Wood units)	3.3 ± 2.2	5.1 ± 3.0	0.070
	Average right atrial pressure (mmHg)	11.4 ± 6.5	14.8 ± 7.2	0.280

Surgical technique employed	Biatrial %	34.4%	65%	0.009

	Any CAV	33.8%	76.2%	<0.001
Cardiac allograft vasculopathy	Significant CAV	25.4%	71.4%	<0.001
	2 or 3 vessels CAV	18.5%	57.1%	<0.001

TR: tricuspid regurgitation; EMBs: endomyocardial biopsies; ACRs: acute cellular rejections; MPAP: mean pulmonary arterial pressure; PVR: pulmonary vascular resistance; CAV: cardiac allograft vasculopathy.

*Data are presented as median and range (min–max).

**According to the revised ISHLT criteria.

**Table 4:** Clinical outcomes of late significant (at least mild-moderate) TR.

Clinical outcome	No TR/mild TR (*n* = 140)	At least mild-moderate TR (*n* = 23)	*P* value
Mortality (% patients)	28.6%	47.8%	0.065
Median serum creatinine at the end of followup (mg/dL)*	1.4 (0.5–10.4)	1.8 (1.0–2.9)	0.081
Need for diuretic therapy ≥ 10 mg furosemide/day (% patients)	10.4%	47.1%	<0.001
Need for another heart surgery	1.7%	33.3%	<0.001

TR: tricuspid regurgitation.

*Data are presented as median and range (min–max).

**Table 5:** Prevalence of TR after OHT.

Study	Number of patients	TR prevalence at the end of the followup (%)	Average follow-up period (years)	Definition of significant TR
Current study	*n* = 163	14.1%	8.2	≥mild-moderate
Chan et al. [3]	*n* = 336	34%	4.5	≥moderate
Aziz et al. [8]	*n* = 249	53.9%	5	≥moderate
Hausen et al. [14]	*n* = 251	50%	4	≥moderate-severe
Williams et al. [18]	*n* = 72	32%	2.4	≥moderate
Yankah et al. [25]	*n* = 647	5.5%	5	≥moderate
Chen et al. [27]	*n* = 178	26.4%	5	≥moderate
Huddleston et al. [28]	*n* = 183	20%	4	≥moderate

TR: tricuspid regurgitation; OHT: orthotopic heart transplantation.
